# *Giardia lamblia*: a major parasitic cause of childhood diarrhoea in patients attending a district hospital in Ghana

**DOI:** 10.1186/1756-3305-4-163

**Published:** 2011-08-22

**Authors:** Bernard Nkrumah, Samuel Blay Nguah

**Affiliations:** 1 Kumasi Centre for Collaborative Research in Tropical Medicine, Kumasi, Ghana; 2 Komfo Anokye Teaching Hospital, Kumasi, Ghana

**Keywords:** *Giardia lamblia*, Intestinal parasites, Rural area, Childhood diarrhoea

## Abstract

**Background:**

Acute childhood diarrhoea remains one of the leading causes of childhood morbidity and mortality in developing countries. The WHO has accordingly underlined the need for epidemiological surveys of infantile diarrhoea in all geographical areas. This study was conducted to determine the incidence of intestinal parasites among stool samples from children examined at a secondary health care facility in a rural area of Ghana.

**Method:**

A retrospective study was conducted to investigate the incidence of intestinal parasites among children who had their stools examined at the Agogo Hospital laboratory. Stool microscopy results from January 2006 through May 2009 were obtained from archived records of the laboratory. Results for children less than 18 years were transcribed unto a standardized datasheet, entered into an electronic database designed using Microsoft^® ^access 2007 and analyzed using *Stata/*SE11.1 statistical software. The incidences of the parasites were determined and presented with their Poisson exact 95% confidence intervals for the various ages.

**Results:**

The median age of the 1080 children included in the study was 5 years (IQR: 2-12 years) with 51.9% being females. The overall incidence of all parasites was 114 per 1000 with *Giardia lamblia *being the most common (89.5%). Children aged less than a year had the lowest parasite incidence of 13 per 1000 with all being *Giardia lamblia*, while those aged 15-17 years had the highest of 169 per 1000. The incidence for *Giardia lamblia *only was lowest at 13 per 1000 for those under a year old, highest at 152 per 1000 for the 15-17 year group and 97 per 1000 for all ages combined. There was a significant rise in incidence of *Giardia lamblia *with age (Trend _x_^2 ^= 18.6, p < 0.001). Five (4.3%) of the 118 positive stool samples had mixed parasites infection. *Enterobius vermicularis*, *Taenia spp *and *Trichuris trichiura *were not seen in any of the stool samples.

**Conclusion:**

*Giardia lamblia *is the most prevalent intestinal parasite in examined stool samples of children within the Ashanti Akim North Municipality and its prevalence significantly increases with age. Measures must be put in place to educate the community on proper personal hygiene to reduce giardiasis.

## Background

Intestinal parasitic infections have a worldwide distribution with high prevalence found in people with low socio-economic status and poor living conditions as well as people in over-crowded areas with poor environmental sanitation, improper garbage disposal, unsafe water supply and unhygienic personal habits [1,2]. These factors are the causes of a major proportion of the burden of disease and death in developing countries [1]. Giardiasis is one of the intestinal protozoa that cause public health problems in most developing countries as well as some developed countries. *Giardia lamblia *is considered to be one of the leading causative agents of diarrhoea in both children [2-4] and adults [5,6]. Many infected persons can be asymptomatic leading to difficulties in the eradication and control of this parasite due to the number of potential carriers such as adult males (5.3%) [7], school children (39.2%) [8] and food vendors (2.0%) [5]. *Giardia lamblia *was observed almost three times more in asymptomatic children (9.7%) than in symptomatic children (3.7%) [9]. Epidemiological surveys have shown that parasitic diarrhoea in children is primarily due to *Giardia lamblia *infection, particularly in areas where fresh vegetables and drinking water sources are contaminated with sewage materials, and foodstuffs can be purchased from street vendors [10]. It has been estimated that about 200 million people are infected each year in Africa, Asia and Latin America [11]. In the industrialized countries, overall prevalence rate of giardiasis is 2-5% [2]. However, in developing countries, *Giardia lamblia *infects children early in life thus a prevalence rate of 15-20% in children younger than 10 years is common. Particularly children who are malnourished are more frequently infected [12]. In Ghana, a number of studies have been published on this subject but these concentrated on particular parasites such as *Entamoeba coli *[13], *Cryptosporidium *[14-16], *Ascaris lumbricoides *[17], *Entamoeba histolytica/Entamoeba dispar *[13] among others [9,17]. The current status of *Giardia lamblia *and other parasitic agents still needs to be evaluated, thus this study was conducted to investigate the incidence of intestinal parasites among stool samples of children in the Ashanti Akim North Municipality over a four year period (2006-2009).

## Methods

### Study site

**Figure 1:**
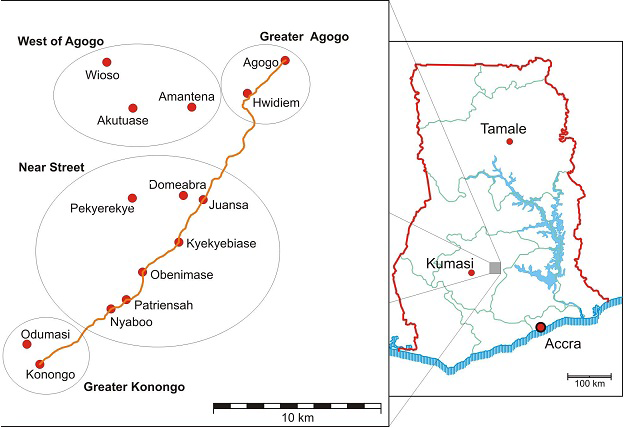
**A map showing the position of the study area and some of the major towns in the Ashanti Akim North Municipality**. Source: Krefis et al., Malaria Journal 2010, 9:201.

The study was conducted at the Agogo Presbyterian Hospital. It is located in the Ashanti Akim North Municipality of the Ashanti Region of Ghana, and is the principal hospital of the municipality and the region. The Ashanti Akim North Municipality is one of the 21 districts in the Ashanti Region. The district is located in the eastern part of Ashanti Region. It covers a land area of 1,160 square kilometers with an estimated population of 170,000 (provisional 2010 population census projection). Over 40% of the population is under 15 years of age and over 50% is under 20 years. Population aged 65 and above make up 6.4% of the total population [18]. The vegetation of the study area is mainly rain forest and the climate is tropical. The temperature variation is between 20 and 36°C with monthly rainfall varying from 2.0 mm in February to 400 mm in July. The major occupation of the people is subsistence farming, animal husbandry and forestry. The sub-districts are Konongo-Odumasi, Agogo, Juansa, Dwease-Praaso and Amanteman (Figure 1). From the Biostatistics Department of the Agogo Hospital, a total of 54,174 outpatient attendances were recorded in the 2007 fiscal year (unpublished data). The top 4 cases reported were acute eye disease (17%), Malaria (15.1%), Upper respiratory tract infections (5.9%) and Diarrhoeal diseases (3.2%). The major sources of water in the district include pipe borne, borehole, stream and well. Environmental Health and Sanitation issues are major problems facing the district.

### Study population and sampling

Archived stool routine examination results at the Agogo Presbyterian Hospital's parasitology laboratory were retrieved. Records of all children less than 18 years and presented to the laboratory for stool parasitological examination from January 2006 to May 2009 were selected out of the lot. Those without verifiable ages were excluded. All children whose stool samples were examined at the laboratory were referred from the Child Welfare Clinic (CWC), pediatric out-patient clinic and the Children's ward of the hospital. Stool analysis results selected were documented on a standardized data collection sheet.

### Stool Collection

The parasitology laboratory of the Agogo Hospital is a well established unit that operates by the internationally accepted Good Laboratory Practice (GLP). Stool samples were collected in accordance with WHO guidelines on the collection of faecal samples [19]. Upon receipt of request from the clinicians, each patient's parent or guardian was given a clean and leak-proof plastic container with an applicator spoon attached to a well fitted screw cup. They were then asked to produce or aid their children to produce stool samples into the container. Otherwise, with the aid of the spoon, they were to transfer a small proportion of the stool into the container and transport it immediately to the sample collection point. At the sample collection point, each sample was labeled with the patient particulars and subject number, and immediately transported into the laboratory with the request form. The average time between sample collection and processing was 20 minutes.

### Sample processing

**Figure 2:**
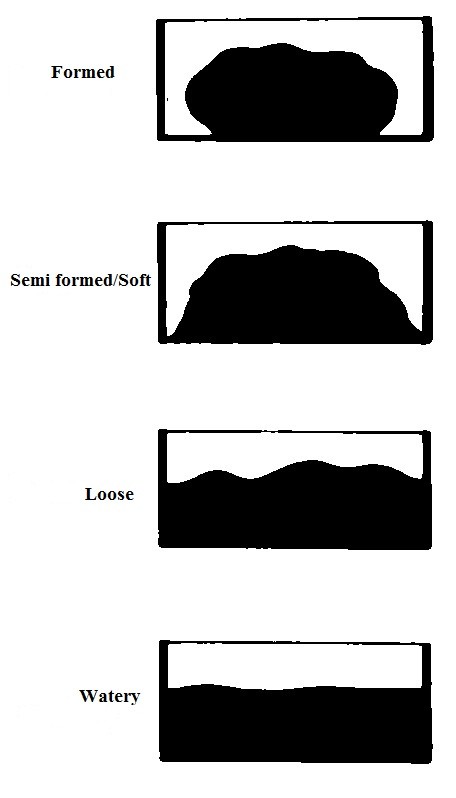
**Macroscopic appearance of stool**. ^¥ ^Mucoid and bloody specimen when observed were recorded.

Stool specimen were examined macroscopically for consistency, mucus and blood. The consistency was reported based on the classification shown in Figure 2. The consistency was used as a guide as to whether the trophozoites or the cyst stage, egg or worm of the parasite was likely to be present (Table 1). When multiple samples were received, mucoid and bloody samples were processed first followed by watery samples.

**Table 1:** Stool categories and techniques applied

		Technique used		
		
Consistency^§^	Parasite stage most likely to seen	Saline	Iodine	Formol Ether
Formed	Cyst	√	√	√
Semi formed/Soft	Cyst/Trophozoites	√	√	√
Loose	Trophozoites	√		√
Watery	Trophozoites	√		√

^§ ^Ova and larvae may be found in stool of any consistency.

Direct microscopy of the smear in saline (0.90% w/v NaCl solution) and Lugol's iodine was performed for the detection of parasites (trophozoites, cysts, egg and larvae) as previously described by Ayeh-Kumi et al. [5]. In cases where parasite species identification was inconclusive the attendant microscopist requested a senior microscopist to confirm the results. Where no consensus was reached, the formol-ether concentration method was employed as previously described [19]. Positive samples were double checked before reports were written and released.

### Data analysis

The transcribed records of the stool samples were double entered into an electronic database designed using Microsoft^® ^access 2007. The data was compared and cleaned for abnormal figures before being transferred to *Stata/*SE 11.1 statistical software (Stata Corporation, Texas USA) for analysis. Categorical variables such as the sex distribution were tabulated and reported with their exact 95% confidence intervals. The incidences of the parasites per 1000 were determined and presented with their Poisson exact 95% confidence intervals stratified for the various age groups. A chi-square test for trend was used to determine the presence of a significant trend in the incidences for the various age groups.

### Ethical Considerations

Ethical approval for the study was obtained from the Committee on Human Research, Publication and Ethics of the School of Medical Sciences, KNUST-Kumasi. Internationally accepted Good Clinical and Good Laboratory practices were applied in all procedures.

## Results

A total of 1080 stool samples from children were retrieved for the study of which majority were females (51.9%). The median age of the children was 5 years (IQR: 2-12 years).

Table 2 gives a detailed description of the samples received and their microscopic outcomes. A majority of the samples received were semi formed (78.4%) whilst bloody samples were the least (0.2%). The presence of red blood cells was recorded in 32 (3.0%, 95% CI: 2.0 - 4.2%) stool samples while white blood cells (Pus cells) were seen in 114 (13.1%, 95% CI: 11.0 - 15.4%).

**Table 2:** Stool macroscopy and presence of parasite

	Parasites present n (%)				
		
Macroscopic stool finding	None	*Giardia lamblia *only	Other Parasites only	Mixed Infection	Total
Bloody	2 (0.2)	0 (0.0)	0 (0.0)	0 (0.0)	2 (0.2)
Formed	7 (0.7)	0 (0.0)	0 (0.0)	0 (0.0)	7 (0.6)
Loose mucoid	14 (1.5)	1 (1.0)	1 (8.3)	1 (20.0)	17 (1.6)
Loose	75 (7.8)	13 (12.9)	1 (8.3)	2 (40.0)	91 (8.4)
Mucoid bloody	4 (0.4)	0 (0.0)	0 (0.0)	0 (0.0)	4 (0.4)
Mucoid	109 (11.3)	0 (0.0)	3 (25.0)	0 (0.0)	112 (10.4)
Semi-formed	751 (78.1)	87 (86.1)	7 (58.3)	2 (40.0)	847 (78.4)

Total	962 (100.0)	101 (100.0)	12 (100.0)	5 (100.0)	1080 (100.0)

One hundred and eighteen (10.9%, 95% CI: 9.1 - 12.9%) of the stool samples were infected, giving an overall incidence of intestinal stool parasites of 114 per 1000 (95% CI: 94.6 - 135.9) (Table 3). *Giardia lamblia*, being the most common intestinal protozoan, was present in 105 infected stool samples (89.0%) followed by *Strongyloides stercoralis *which was present in 7 samples (5.9%). *Hookworm *was seen in 4 samples (3.4%), *Entamoeba histolytica/Entamoeba dispar *in 2 samples (1.7%), and *Schistosoma mansoni *in 1 sample (0.8%).

**Table 3:** Incidence (per 1000) of intestinal parasites in stool samples for various age groups

	Age Group Incidence (95% CI)						
	
	< 1 yr	1-2 yrs	3-5 yrs	6-10 yrs	11-14 yrs	15-17 yrs	Overall
Parasite	(n = 227)	(n = 151)	(n = 189)	(n = 191)	(n = 145)	(n = 177)	(n = 1080)
*G. lamblia*	13 (2.7 - 38.6)	99 (55.6 - 163.8)	138 (89.9 - 201.6)	89 (51.8 - 142.5)	117 (68.3 - 187.7)	152 (100.5 - 221.9)	97 (79.5 - 117.7)
*E. coli*	0 (0.0 - 16.2)	0 (0.0-24.4)	5.3 (0.1-29.5)	0 (0.0-19.3)	6.9 (0.1-38.4)	0 (0.0-20.8)	2 (0.2 - 6.7)
*Other flagellates*	0 (0.0 - 16.2)	6.6 (0.2-36.9)	0 (0.0-19.5)	0 (0.0-19.3)	0 (0.0-25.4)	0 (0.0-20.8)	1 (0.0 - 5.2)
*H. nana*	0 (0.0 - 16.2)	6.6 (0.2-36.9)	0 (0.0-19.5)	0 (0.0-19.3)	0 (0.0-25.4)	0 (0.0-20.8)	1 (0.0 - 5.2)
*Hookworm*	0 (0.0 - 16.2)	0 (0.0-24.4)	0 (0.0-19.5)	5.2 (0.1-29.2)	13.8 (1.7-49.8)	5.6 (0.1-31.5)	4 (1.0 - 9.5)
*S. stercoralis *	0 (0.0 - 16.2)	13.2 (1.6-47.8)	5.3 (0.1-29.5)	5.2 (0.1-29.2)	6.9 (0.1-38.4)	11.3 (1.4-40.8)	6 (2.6 - 13.3)
*E. histolytica/dispar*	0 (0.0 - 16.2)	6.6 (0.2-36.9)	5.3 (0.1-29.5)	0 (0.0-19.3)	0 (0.0-25.4)	0 (0.0-20.8)	2 (0.2 - 6.7)
*S. mansoni*	0 (0.0 - 16.2)	0 (0.0-24.4)	0 (0.0-19.5)	5.2 (0.1-29.2)	0 (0.0-25.4)	0 (0.0-20.8)	1 (0.0 - 5.1)

The incidence per 1000 of the various intestinal parasites were; *Giardia lamblia *97.2 (95% CI: 79.5 - 117.7), *Strongyloides stercoralis *6 (95% CI: 2.6 - 13.3), Hookworm 4 (95% CI: 1.0 - 9.5), *Entamoeba histolytica/Entamoeba dispar *2 (95% CI: 0.2 - 6.7) and *Entamoeba coli (E. coli) *2 (95% CI: 0.2 - 6.7). *Hymenolepis nana*, other intestinal flagellates and *Schistosoma mansoni *all had incidences of 1 per 1000 (95% CI: 0.0 - 5.2). *Enterobius vermicularis*, *Taenia spp *and *Trichuris trichiura *were not seen in any of the stool samples.

**Figure 3:**
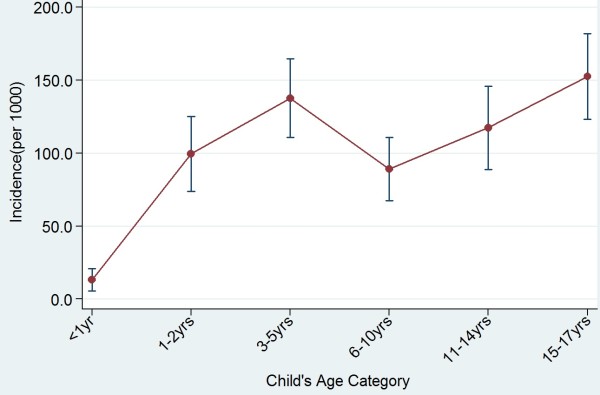
**Incidence (± se) of *Giardia lamblia *in children**.

*Giardia lamblia *was seen almost exclusively in stools with either a loose or semi-formed consistency. There was a significant rise in its presence with age (Trend _x_^2 ^= 18.6, p < 0.001) (Figure 3).

Mixed parasitic infections were seen in 5 (4.2%, 95% CI: 1.4 - 9.6%) of the infected stool samples. Two of the mixed infections were *Giardia lamblia *and *Entamoeba coli *co-infections, one *Giardia lamblia *and hookworm, one *Giardia lamblia *and *Strongyloides stercoralis*, and the last one was *Hymenolepis nana *and other flagellate co-infection.

## Discussion

People who generally reside in rural or under developed areas are more prone to the ingestion of infective parasites as compared to those who live in urban/suburban or well developed areas where sanitation is presumably better; hence possess a lower chance of infection [8]. The water supply in developed areas is obviously cleaner, which reduces the chance of contamination. However in rural areas, the nature of everyday activities bring people, especially children, into close contact with natural sources of soil and water, therefore increasing their risk of ingestion as well as the penetration of infective stage parasites [4,16].

10.9% of children's stool samples examined in this study were found to have intestinal parasites. This study result is in agreement with studies carried out earlier in diarrheal children in Kumasi, Ghana (11.0%) [14] and pre-school children in Gaza, Palestine (10.3%). Both studies showed no clear trend in prevalence with age [20]. Studies conducted by Annan et al among pre-school children in Ghana however revealed up to 18.2% *Giardia *infection [17] whilst Verweij et al reported 21.5% among children in Northern Ghana [13]. Even higher prevalence have been reported in other areas such as among children in the urban slums of Karachi, Pakistan (23.9%), Iranian day-care children (26.2%) [21], children in the aborigine community in Pahang, Malaysia (44.1%) [2], and children in Amman, Jordan (78%) [22].

The lower prevalence of *Giardia lamblia *in our study could be attributed mainly to the technique employed (Direct wet mount) in the identification of the parasites as compared to the techniques employed in the other studies. The direct wet mount technique is fast, cheap and easy for the diagnosis of intestinal parasites when present in sufficient concentrations [23]. It detects motility of organisms [24] and valuable for detection of parasites that may be lost in the concentration methods [25] as well as the examination of certain diagnostically important objects such as cellular exudates [26,27]. However, this method lacks sensitivity [23,25,28-30] and its parasite detection ability is even lower at low parasite concentrations for even the best of microscopists [31,32]. Slide preparations from wet mounts dry up easily thus motile organisms may not be detected if the preparations are not examined quickly after preparation [33].

In contrast to other studies conducted among infants in Kumasi, Ghana [4], among children in northern Ghana [9], children in Lagos, Nigeria [34], Côte d'Ivoire [35], Qatar [36] and in Delhi, India [37], this study showed a high prevalence of *Giardia lamblia *in children. This suggests that *Giardia lamblia *infection may either be present sub-clinically or the parasite have partial pathogenicity or the majority of the children within the study area are asymptomatic carriers of a non-pathogenic strain. Different genotypes of *Giardia lamblia *(Assemblage A and Assemblage B) has been reported in Bangladesh with the Assemblage A genotype more associated with diarrhoea than the Assemblage B genotype [38].

*Giardia lamblia *has been documented to be transmitted either from person to person, animal to person or from the environment to person. These transmission modes are well favored by high temperatures and moist climatic conditions, poor personal hygiene and unsanitary habits of individuals [2,5,8,37]. Again, domestic animals such as dogs which serve as reservoir hosts for *Giardia lamblia *provide the utmost risk of the infection [39]. The study area as earlier described possesses these conditions that are favorable for the transmission of *Giardia lamblia *and other parasitic agents. With subsistence farming and animal husbandry being the major occupation of the people, most households have domestic animals such as dogs, sheep, goats, etc which are often allowed to roam outdoors either unsupervised or in the company of children. Due to lack of potable water on their farms, the farmers and their children drink from streams and rivers which are sometimes used by these animals also. These factors might have contributed to the high prevalence rate of *Giardia lamblia *infection in children within the study area.

*Giardia lamblia *incidence increased significantly with age (Trend ^2^= 18.6, p < 0.001) with the highest age group being 15-17 years (Figure 3). Majority of the patients in this study were children of school age and thus they have very active playing habits in and out of school. These children normally play in the soil which harbors these parasites and are less mindful of some very important personal hygiene practices such as washing of hands with soap and water before eating, after playing in the soil and after visiting the toilets. Again, they also buy a lot of food from streets vendors some of whom do not practice proper personal hygiene and may also be carriers of some of these infective parasites [5,6]. It has also been shown that children acquire immunity after the initial infections in early life which results in some protection in later life [40]. This study shows that children of school going age are also highly affected by giardiasis contrary to previous suggestions that giardiasis was highest only among children of pre-school age who are usually in child care settings [21].

## Conclusion

*Giardia lamblia *is the most prevalent intestinal parasite among children within the Ashanti Akim North Municipality. Measures must be put in place to educate the community and the children on proper personal hygiene to reduce giardiasis.

## List of abbreviations

IQR: Interquartile Range; CI: Confidence Interval; KNUST: Kwame Nkrumah University of Science and Technology; UPO: University Post Office; mm: millimeter; w/v: weight per volume; NaCl: Sodium Chloride; USA: United States of America; °C: Degree Celsius; SE: Standard Edition; WHO: World Health Organization

## Competing interests

The authors declare that they have no competing interests.

## Authors' contributions

BN planned and designed the study protocol, carried out the study and headed the writing of the manuscript. SBN designed the study protocol, performed the statistical analysis and contributed to the writing of the manuscript. All the authors have read and approved the manuscript.
